# Mitoxyperilysis Drives Immunosuppressive Tumor Microenvironment Remodeling and Hepatocellular Carcinoma Progression via ANXA1-Mediated Macrophage M2 Polarization

**DOI:** 10.3390/cancers18172786

**Published:** 2026-08-27

**Authors:** Meng Qin, Wenzhi He, Gang Wu

**Affiliations:** 1 General Surgery, Tianyou Hospital, Wuhan University of Science and Technology, Wuhan 430071, China; 2025283030147@whu.edu.cn; 2 Department of Hepatobiliary and Pancreatic Surgery, Zhongnan Hospital of Wuhan University, Wuhan 430071, China

**Keywords:** mitoxyperilysis, hepatocellular carcinoma, tumor microenvironment, ANXA1, macrophage M2 polarization

## Abstract

Background: Mitoxyperilysis is a recently described form of regulated cell death, but its clinical and immunological relevance in hepatocellular carcinoma (HCC) remains unclear. This study investigated the prognostic significance of mitoxyperilysis-related transcriptional activity and its role in remodeling the tumor immune microenvironment. Methods: A Mitoxyperilysis Score was generated by single-sample gene set enrichment analysis. Prognostic associations were evaluated using Kaplan–Meier analysis, multivariable Cox regression, independent cohort validation, and random-effects meta-analysis. Immune infiltration and cell–cell communication were examined using bulk transcriptomic and single-cell RNA-sequencing data. The functional role of ANXA1 was assessed by ELISA, Western blotting, macrophage co-culture, immunofluorescence, flow cytometry, *ANXA1* knockdown, and subcutaneous tumor models. Results: High Mitoxyperilysis Scores were associated with shorter overall survival in TCGA-LIHC (log-rank *p* = 0.032), although the association was attenuated after adjustment for age, sex, stage, grade, and vascular invasion. The adverse survival association was independently reproduced in the Gao2019 CHCC-HBV cohort (log-rank *p* = 0.00383). Across TCGA-LIHC, GSE14520, and Gao2019 CHCC-HBV, a random-effects analysis showed an overall hazard ratio of 1.42 per 1-standard-deviation increase in score (95% CI, 1.08–1.86; *p* = 0.011), with substantial between-cohort heterogeneity (*I*^2^ = 77.5%). High-score tumors displayed increased immune and stromal infiltration, immune-checkpoint expression, and M2-like macrophage enrichment. *ANXA1* expression correlated positively with the Mitoxyperilysis Score in independent cohorts. Mitoxyperilysis induction increased both intracellular and extracellular ANXA1, while ANXA1 overexpression promoted CD163^+^CD206^+^ macrophage polarization. ANXA1 knockdown attenuated this response, reduced subcutaneous tumor burden, and decreased intratumoral CD163 staining. Conclusions: Mitoxyperilysis-related transcriptional activity is associated with adverse survival and an M2-enriched immunosuppressive microenvironment in HCC. ANXA1 is an important, although probably not exclusive, mediator of this process and may represent a potential therapeutic target.

## Simple Summary

Primary liver cancer remains difficult to treat because tumor cells can reshape their surroundings and weaken immune responses. This study examined mitoxyperilysis, a newly described form of cell death, to determine whether it contributes to this immune suppression. By analyzing patient gene-expression data and testing liver cancer cells in laboratory and mouse models, we found that tumors with stronger mitoxyperilysis-related signals contained more tumor-supporting macrophages and were linked to poorer outcomes. The cancer cells also released more Annexin A1, which promoted macrophages toward an immune-suppressive state, while reducing Annexin A1 weakened this effect and slowed tumor growth. These findings suggest that the connection between mitoxyperilysis, Annexin A1, and macrophages may help explain immune escape in liver cancer and may guide future biomarker and treatment research.

## 1. Introduction

Hepatocellular carcinoma (HCC) constitutes the predominant histological subtype of primary liver malignancy and ranks among the leading causes of cancer-related mortality on a global scale, with an estimated annual incidence exceeding 900,000 new cases and a five-year survival rate that remains dismally below 20% for patients presenting with advanced disease [1,2]. Despite incremental therapeutic advances achieved through the integration of targeted molecular agents and immune checkpoint blockade into clinical practice, the prognosis of HCC remains profoundly unsatisfactory, a circumstance attributable in substantial measure to the extraordinary cellular and molecular heterogeneity of this tumor type, the frequency of late-stage diagnosis, and the propensity for resistance to systemic therapies [3,4]. In this context, a fundamental imperative exists to elucidate novel biological mechanisms that govern HCC progression, with particular emphasis on processes capable of remodeling the immunological landscape of the tumor microenvironment (TME).

Regulated cell death (RCD) has progressively transcended its classical conceptualization as a mere homeostatic mechanism, emerging instead as a pivotal determinant of tumor biology that profoundly influences immune surveillance, microenvironmental composition, and therapeutic responsiveness [5,6]. Over the preceding decade, a succession of molecularly distinct RCD modalities—including apoptosis, necroptosis, pyroptosis, ferroptosis, cuproptosis, and disulfidptosis—have been characterized at the mechanistic level, each exhibiting unique immunological footprints and context-dependent consequences for tumor immune evasion or activation [7,8,9,10]. Accumulating evidence suggests that the immunogenicity associated with specific RCD modalities critically shapes the balance between anti-tumor immunity and immunosuppressive microenvironmental programming, thereby influencing disease trajectory and responsiveness to immunotherapy [11,12]. Nevertheless, the biological repertoire of RCD remains incompletely catalogued, and the functional implications of newly described death modalities in cancer contexts are frequently undefined.

Mitoxyperilysis represents a recently identified form of regulated cell death distinguished by a characteristic molecular signature encompassing mediators of mitochondrial integrity surveillance, innate immune pattern recognition, and cytoskeletal regulatory networks, including *BAK1*, *BAX*, *BID*, *MYD88*, *RHOA*, *RICTOR*, *TLR2*, *TLR4*, *TLR7*, *TLR8*, *TNFRSF1A*, and *TNFRSF1B*. Conceptually, this death modality appears to integrate mitochondria-dependent apoptotic licensing with innate immune receptor co-activation and mechanosensory signaling, potentially generating an immunologically distinctive cell death milieu. However, whether and how mitoxyperilysis activity influences the transcriptional landscape, immunological composition, and clinical outcomes of HCC have not been systematically investigated.

The TME of HCC is characterized by remarkable immunosuppressive complexity, wherein tumor-associated macrophages (TAMs) occupy a particularly influential niche [13,14]. Recent evidence further indicates that the immune complexity of HCC is closely intertwined with aberrant vascular remodeling and stromal activation. Dynamic crosstalk among malignant cells, immune populations, cancer-associated fibroblasts, hepatic stellate cells, and vascular endothelial cells collectively promotes immune evasion, angiogenesis, and resistance to systemic therapies. This interconnected immune–stromal–vascular network highlights that macrophage polarization occurs within a broader multicellular ecosystem rather than as an isolated event [15]. TAMs in HCC predominantly adopt an M2-like polarization state associated with pro-tumorigenic functions encompassing promotion of angiogenesis, suppression of cytotoxic lymphocyte activity, remodeling of extracellular matrix, and secretion of immunomodulatory cytokines that collectively reinforce immune exclusion and facilitate metastatic dissemination [16,17]. The molecular signals emanating from tumor cells that instruct this macrophage polarization program remain an area of intensive investigation, as their identification may reveal targetable axes for combination immunotherapy strategies [18].

Annexin A1 (ANXA1), a calcium-dependent phospholipid-binding protein with established anti-inflammatory and membrane repair functions, has recently garnered attention as a mediator of tumor-immune interactions through its engagement with formyl peptide receptors (FPRs) expressed on myeloid cells [19,20]. ANXA1-FPR signaling has been implicated in diverse immunomodulatory contexts, yet its potential role in transducing signals from dying or stressed tumor cells to macrophages within the HCC microenvironment has not been elucidated.

In the present study, we undertook a comprehensive, multi-dimensional investigation to define the biological significance of mitoxyperilysis in HCC. Through integrated transcriptomic profiling of TCGA-LIHC and GSE14520 cohorts, we systematically characterized the expression landscape of mitoxyperilysis-associated genes, established their prognostic relevance, and delineated downstream transcriptional and immunological consequences of differential mitoxyperilysis activity. Leveraging immune deconvolution algorithms [21], ESTIMATE-based microenvironmental scoring [22], and single-cell intercellular communication analysis, we identified M2 macrophage polarization and ANXA1-FPR signaling as cardinal downstream consequences of elevated mitoxyperilysis activity. These computational observations were experimentally substantiated through in vitro co-culture models, demonstrating that mitoxyperilysis-induced hepatoma cells actively drive macrophage M2 skewing via ANXA1 upregulation [23]. Taken together, our findings unveil a previously undescribed immunosuppressive circuit initiated by mitoxyperilysis in HCC, providing a mechanistic framework with potential implications for biomarker development and therapeutic targeting.

## 2. Materials and Methods

### 2.1. Ethical Statement

The analyses of human data were based exclusively on de-identified, publicly available datasets and therefore did not require additional institutional review or informed consent. All animal experiments were conducted in accordance with institutional guidelines for the care and use of laboratory animals and were approved by the Institutional Animal Care and Use Committee of Zhongnan Hospital of Wuhan University (approval code: Kelun-2025477; approved on 17 March 2025).

### 2.2. Public Dataset Acquisition and Preprocessing

Transcriptomic expression data and matched clinical annotations for HCC were obtained from two independent publicly accessible repositories. The primary discovery cohort comprised RNA-sequencing profiles (log_2_ RSEM-normalized counts) alongside corresponding clinical metadata from The Cancer Genome Atlas Liver Hepatocellular Carcinoma project (TCGA-LIHC), accessed via the UCSC Xena browser (https://xenabrowser.net; accessed on 10 March 2026). The TCGA-LIHC cohort comprised 354 primary HCC specimens and 50 non-tumorous liver specimens classified by the Genomic Data Commons as “Solid Tissue Normal” (TCGA sample type code 11). These comparator specimens represented non-neoplastic liver tissues collected from patients with HCC, generally from regions adjacent to the tumor, rather than healthy liver tissues obtained from cancer-free donors. Because information on fibrosis and cirrhosis was not consistently available for all 50 specimens, they could not be reliably categorized as cirrhotic or non-cirrhotic and were therefore analyzed collectively as adjacent non-tumorous liver tissues. An independent validation cohort was retrieved from the Gene Expression Omnibus (GEO) repository under accession number GSE14520 (https://www.ncbi.nlm.nih.gov/geo/; accessed on 10 March 2026), which contains microarray-based expression profiles generated on the Affymetrix HG-U133A platform from a Chinese HCC patient population. For the GSE14520 dataset, probe-level intensities were subjected to robust multi-array average (RMA) normalization, and genes represented by multiple probe sets were collapsed to a single value by retaining the probe with the highest mean expression across all samples. Additionally, expression and survival data from the International Cancer Genome Consortium (ICGC) (https://dcc.icgc.org/; accessed on 10 March 2026) liver cancer cohort were incorporated to facilitate cross-platform correlation validation. All datasets were accessed in compliance with their respective data usage policies, and no additional ethical approvals were required given their de-identified, publicly available nature.

### 2.3. Mitoxyperilysis Gene Set Definition and Activity Scoring

A twelve-gene panel was manually curated from molecular components implicated in the original mechanistic study of mitoxyperilysis [24]. The panel comprised *BAK1*, *BAX*, *BID*, *MYD88*, *RHOA*, *RICTOR*, *TLR2*, *TLR4*, *TLR7*, *TLR8*, *TNFRSF1A*, and *TNFRSF1B*. To derive a continuous sample-level score, single-sample gene set enrichment analysis (ssGSEA) was applied to the normalized expression matrices using the GSVA package. The resulting score was termed the “Mitoxyperilysis-Related Gene Signature Score” and was interpreted as an exploratory transcriptomic proxy rather than a validated or exclusively specific indicator of mitoxyperilysis execution. To derive a continuous, sample-level quantification of mitoxyperilysis transcriptional activity, single-sample Gene Set Enrichment Analysis (ssGSEA) was applied to the normalized expression matrices of all cohorts using the GSVA package (version 1.42.0) implemented in R (version 4.3.1). The ssGSEA algorithm computes an enrichment score for each sample independently by integrating the rank-based distribution of gene set members relative to all expressed genes, thereby generating a score that is interpretable across samples without requiring group-level comparisons. The resulting per-sample ssGSEA enrichment scores were designated as the “Mitoxyperilysis Score” and employed as the primary quantitative metric throughout all downstream analyses. Patients were subsequently stratified into high- and low-mitoxyperilysis groups using an optimal cutoff determined by maximally selected rank statistics, as implemented in the survminer package (version 0.4.9).

### 2.4. Differential Expression and Transcriptomic Landscape Analysis

To characterize transcriptional programs associated with divergent mitoxyperilysis activity, differential gene expression analysis was conducted between high- and low-mitoxyperilysis patient subgroups within the TCGA-LIHC tumor sample set. Differential expression was computed using the limma package (version 3.56.2) with empirical Bayes moderation of standard errors, applying voom transformation to account for mean–variance relationships in count-derived data. Genes satisfying thresholds of |log_2_ fold change| > 1.0 and Benjamini–Hochberg adjusted *p*-value < 0.05 were designated as differentially expressed genes (DEGs). Unsupervised dimensionality reduction was performed via principal component analysis (PCA) to visualize transcriptome-wide separation between patient subgroups. The top-ranked DEGs were visualized as volcano plots and hierarchically clustered heatmaps using the pheatmap package (version 1.0.12) with ward. D2 linkage and Euclidean distance metrics.

### 2.5. Functional Enrichment Analysis

To systematically interpret the biological pathways associated with high mitoxyperilysis activity, both over-representation analysis (ORA) and gene set enrichment analysis (GSEA) were performed using the clusterProfiler package (version 4.6.0) in R. For ORA, the complete ranked DEG list was interrogated against Gene Ontology Biological Process (GO-BP) terms and Kyoto Encyclopedia of Genes and Genomes (KEGG) pathway annotations. Enrichment significance was evaluated using a hypergeometric test, with pathway terms satisfying adjusted *p*-value < 0.05 and a minimum gene count threshold of 5 considered statistically significant. For GSEA, all expressed genes were pre-ranked according to the signed −log_10_ (adjusted *p*-value) metric derived from the differential expression analysis, and canonical KEGG gene sets were used as reference collections. A normalized enrichment score (NES) with 1000 permutations and false discovery rate (FDR) < 0.25 defined significant enrichment. Results were visualized as bubble plots and running enrichment score curves.

### 2.6. ESTIMATE Algorithm

Stromal and immune infiltration levels within each tumor sample were quantitatively estimated using the ESTIMATE algorithm, which leverages two curated gene signatures—one reflective of non-malignant stromal cell content and one reflective of immune cell infiltration—to generate three composite scores per sample: the Stromal Score, Immune Score, and ESTIMATE Score (the arithmetic sum of the former two). Analyses were performed using the estimate R package (version 1.0.13) applied to TCGA-LIHC normalized expression data. Spearman correlation coefficients between ESTIMATE-derived scores and the continuous Mitoxyperilysis Score were computed to quantify the linear association between mitoxyperilysis activity and microenvironmental composition.

### 2.7. Immune Checkpoint Molecule Profiling

Expression levels of twelve canonical immune checkpoint and co-stimulatory molecules—including *CD274* (PD-L1), *CD28*, *CD80*, *CD86*, *CTLA4*, *HAVCR2* (TIM-3), *ICOS*, *IDO1*, *LAG3*, *PDCD1* (PD-1), *PDCD1LG2* (PD-L2), and *TIGIT*—were extracted from the TCGA-LIHC expression matrix and compared between high- and low-mitoxyperilysis groups using the Wilcoxon rank-sum test. Spearman correlation between each immune checkpoint transcript and the Mitoxyperilysis Score was subsequently computed across the full cohort, and significance was assessed with Bonferroni correction for multiple comparisons. Results were presented as grouped box plots, hierarchically clustered heatmaps, and a dot-matrix correlation overview panel.

### 2.8. ssGSEA-Based Immune Profiling

The fractional representation of 23 distinct immune cell populations was estimated across TCGA-LIHC and GSE14520 using ssGSEA with a curated immune cell signature compendium. Enrichment scores for each immune subset were computed per sample and compared between high- and low-mitoxyperilysis groups using the Wilcoxon rank-sum test with FDR correction.

### 2.9. CIBERSORT-like Deconvolution

Complementary immune cell fraction estimation was performed using a CIBERSORT-like computational pipeline with the LM22 immune cell reference matrix, which deconvolves bulk transcriptomic data into 22 distinct immune cell subpopulations through constrained quadratic programming. Analyses were executed in both the TCGA-LIHC and GSE14520 cohorts independently. Differences in estimated cell fractions between mitoxyperilysis subgroups were assessed by Wilcoxon rank-sum testing, and results were presented as grouped box plots with annotated significance levels. Cell types demonstrating concordant directional changes across both independent cohorts were designated as robustly associated with mitoxyperilysis activity.

### 2.10. Single-Cell RNA Sequencing Data Processing and Cell–Cell Communication Analysis

Processed single-cell RNA-sequencing data were obtained from the Gene Expression Omnibus under accession number GSE149614, originally reported by Lu et al. [25]. The dataset comprised 71,915 cells from 21 specimens obtained from 10 patients with hepatocellular carcinoma, including 10 primary tumors, eight non-tumor liver tissues, two portal vein tumor thrombi, and one metastatic lymph node. In the original study, freshly resected tissues were mechanically minced, enzymatically dissociated, filtered into single-cell suspensions, and subjected to single-cell RNA sequencing using the 10× Genomics Chromium platform (10× Genomics, Pleasanton, CA, USA). Raw count matrices were imported into R and analyzed using the Seurat framework (version 4.3.0). Standard quality control criteria were applied, retaining cells with 200–6000 detected genes, fewer than 25% mitochondrial read fractions, and total UMI counts within established interquartile range thresholds. Data were normalized using the NormalizeData function (log normalization, scale factor = 10,000), followed by identification of highly variable genes, PCA-based dimensionality reduction, and graph-based clustering via the FindClusters function (resolution = 0.5). Cell type identities were assigned through canonical marker gene expression and visualized using Uniform Manifold Approximation and Projection (UMAP).

Hepatocyte clusters were further sub-stratified into mitoxyperilysis-high and mitoxyperilysis-low populations based on the per-cell ssGSEA enrichment scores computed for the mitoxyperilysis gene set, with the median score serving as the stratification threshold. Differential intercellular communication between these cancer cell subpopulations and the myeloid cell compartment was interrogated using the CellChat package (version 1.6.0). Ligand–receptor interaction probabilities were computed based on the law of mass action using curated interaction databases, and differential communication networks between Cancer_Mitoxy_High → Myeloid and Cancer_Mitoxy_Low → Myeloid axes were identified by comparing interaction number, strength, and specific ligand–receptor pairs. Signaling pathways demonstrating significant and directionally consistent enrichment in the high-mitoxyperilysis cancer subpopulation were prioritized for subsequent mechanistic investigation.

### 2.11. ANXA1 Expression Analysis and Correlation with Mitoxyperilysis

To evaluate the transcriptional relationship between *ANXA1* and mitoxyperilysis activity at the population level, Spearman correlation analysis was conducted between the continuous Mitoxyperilysis Score and *ANXA1* mRNA expression across three independent cohorts: TCGA-LIHC, ICGC, and GSE14520. Correlation coefficients (R) and associated *p*-values were computed, and scatter plots with fitted regression lines and 95% confidence intervals were generated. Additionally, *ANXA1* expression levels were compared between high- and low-mitoxyperilysis patient subgroups in TCGA-LIHC *(n* = 184 versus 185) and ICGC (*n* = 116 versus 116) using the Wilcoxon rank-sum test.

### 2.12. Cell Culture and Induction of Mitoxyperilysis

Human hepatocellular carcinoma Huh-7 cells were obtained from the China Center for Type Culture Collection (CCTCC), Wuhan University, Wuhan, China (catalogue no. GDC0134; RRID: CVCL_0336). Cells were maintained in Dulbecco’s Modified Eagle Medium (DMEM) supplemented with 10% heat-inactivated fetal bovine serum and 1% penicillin–streptomycin at 37 °C in a humidified atmosphere containing 5% CO_2_. Human THP-1 monocytic cells were cultured in RPMI-1640 medium supplemented with 10% FBS and differentiated into macrophages by treatment with 100 nM phorbol 12-myristate 13-acetate (PMA) for 48 h prior to experimental use.

To establish a mitoxyperilysis-inducing condition, Huh-7 cells were washed once with phosphate-buffered saline and transferred to carbon-starvation DMEM lacking glucose, L-glutamine, and sodium pyruvate (Gibco, Life Technologies Corporation, Grand Island, NY, USA; catalogue no. A14430-01). Ultrapure lipopolysaccharide derived from *Escherichia coli* O111:B4 (InvivoGen, San Diego, CA, USA; catalogue no. tlrl-3pelps) was added at a final concentration of 200 EU/mL, and the cells were incubated for 24 h. This treatment condition was adapted from the combined innate immune activation and metabolic disruption protocol described by Wang et al. Parallel controls included cells maintained in complete DMEM, cells cultured in carbon-starvation medium alone, and cells treated with 200 EU/mL LPS in complete DMEM. After treatment, the expression levels of ANXA1, GPX4, HSP60, COX IV, and TOM20 were examined by Western blotting, with β-actin used as the loading control. These proteins were evaluated as supportive biochemical measurements under the mitoxyperilysis-inducing condition rather than as specific markers independently confirming mitoxyperilysis [24].

For ANXA1 overexpression experiments, HCC cells were transfected with a full-length human *ANXA1* expression construct cloned into a mammalian overexpression vector (pCMV backbone), or an empty vector control (Vector), using Lipofectamine 3000 transfection reagent (Thermo Fisher Scientific, Waltham, MA, USA). Overexpression efficiency was validated by Western blotting 48 h post-transfection.

### 2.13. Macrophage Co-Culture System and M2 Polarization Assessment

To evaluate the paracrine capacity of mitoxyperilysis-induced HCC cells to promote macrophage polarization, a transwell-based indirect co-culture system was established. PMA-differentiated THP-1-derived macrophages were seeded in the lower chamber of 0.4-μm pore transwell inserts (Corning Incorporated, Corning, NY, USA), while treated HCC cells (either mitoxyperilysis-induced or ANXA1-overexpressing, with respective controls) were placed in the upper compartment. Co-cultures were maintained for 48 h under standard conditions, after which macrophages were harvested for phenotypic characterization.

### 2.14. Immunofluorescence Staining

Macrophages recovered from co-culture experiments were fixed with 4% paraformaldehyde for 15 min at room temperature, permeabilized with 0.1% Triton X-100, and blocked with 5% normal goat serum for 1 h. Cells were subsequently incubated overnight at 4 °C with a primary antibody targeting CD206 (Mannose Receptor; anti-human, rabbit monoclonal), followed by incubation with an Alexa Fluor 488-conjugated secondary antibody for 1 h at room temperature protected from light. Nuclei were counterstained with DAPI (4′,6-diamidino-2-phenylindole). Fluorescence images were acquired using a confocal laser scanning microscope (Leica Microsystems, Wetzlar, Germany) at standardized exposure settings, and relative CD206 fluorescence intensity was quantified using ImageJ software (version 1.54f, National Institutes of Health, Bethesda, MD, USA) (NIH) by analyzing a minimum of five randomly selected fields per condition.

### 2.15. Flow Cytometry

M2 macrophage polarization was quantified by multiparameter flow cytometry using surface markers CD163 and CD206, which together define the canonical M2-like phenotype. Following co-culture, macrophages were detached, washed twice with phosphate-buffered saline (PBS) containing 2% FBS, and incubated with fluorochrome-conjugated antibodies against CD163 (PE-labeled) and CD206 (APC-labeled) for 30 min at 4 °C in the dark. Cells were subsequently washed, resuspended in PBS, and analyzed using a BD FACSCanto II flow cytometer (BD Biosciences, San Jose, CA, USA). Data acquisition targeted a minimum of 10,000 events per sample, and analysis was performed using FlowJo software (version 10.8, BD Biosciences). The M2 macrophage proportion was defined as the percentage of CD163^+^CD206^+^ double-positive cells within the gated macrophage population.

### 2.16. Western Blotting

Protein lysates were prepared by incubating cell pellets in RIPA lysis buffer supplemented with protease and phosphatase inhibitor cocktails (Sigma-Aldrich, St. Louis, MO, USA) on ice for 30 min, followed by centrifugation at 12,000× *g* for 15 min at 4 °C to remove insoluble debris. Protein concentrations were quantified using the BCA protein assay kit (Thermo Fisher Scientific, Waltham, MA, USA). Equal quantities of protein (30 μg per lane) were resolved by SDS-polyacrylamide gel electrophoresis on 10–12% gradient gels and subsequently transferred onto polyvinylidene difluoride (PVDF) membranes (EMD Millipore, Burlington, MA, USA) at 100 V for 90 min. Membranes were blocked with 5% non-fat dry milk dissolved in Tris-buffered saline containing 0.1% Tween-20 (TBST) for 1 h at room temperature and then incubated overnight at 4 °C with primary antibodies targeting ANXA1, GPX4, HSP60, COX IV, TOM20, and β-actin. Following three washes with TBST, membranes were incubated with horseradish peroxidase-conjugated secondary antibodies for 1 h at room temperature. Protein bands were visualized using enhanced chemiluminescence (ECL) reagent and imaged using a ChemiDoc imaging system (Bio-Rad Laboratories, Hercules, CA, USA). Band intensities were quantified densitometrically using ImageJ (version 1.54f, National Institutes of Health, Bethesda, MD, USA), with normalization to the β-actin loading control. Detailed information on all antibodies used for Western blotting, immunofluorescence, and flow cytometry, including catalog numbers, clone information, host species, conjugates, and working dilutions, is provided in Table S1.

### 2.17. Statistical Analysis

All statistical computations were conducted within the R software environment (version 4.3.1). Comparative analyses between two independent groups were performed using the two-tailed Wilcoxon rank-sum test, whereas comparisons across three or more groups employed the Kruskal–Wallis test followed by Dunn’s post-hoc analysis with Bonferroni correction. Spearman’s rank correlation was applied to evaluate monotonic associations between continuous variables. Overall survival analysis was conducted using the Kaplan–Meier method, with survival curves compared by the log-rank test; the optimal stratification cutoff was identified using maximally selected rank statistics from the survminer package (version 0.4.9). All *p*-values were two-tailed, and statistical significance was defined as *p* < 0.05. Multiple testing corrections were applied where indicated using the Benjamini–Hochberg FDR method. Graphical outputs were generated using ggplot2 (version 3.4.2) and pheatmap (version 1.0.12) within the R environment.

## 3. Results

### 3.1. Mitoxyperilysis-Related Transcriptional Activity Is Associated with Clinical Outcome Across Independent HCC Cohorts

**Figure 1:**
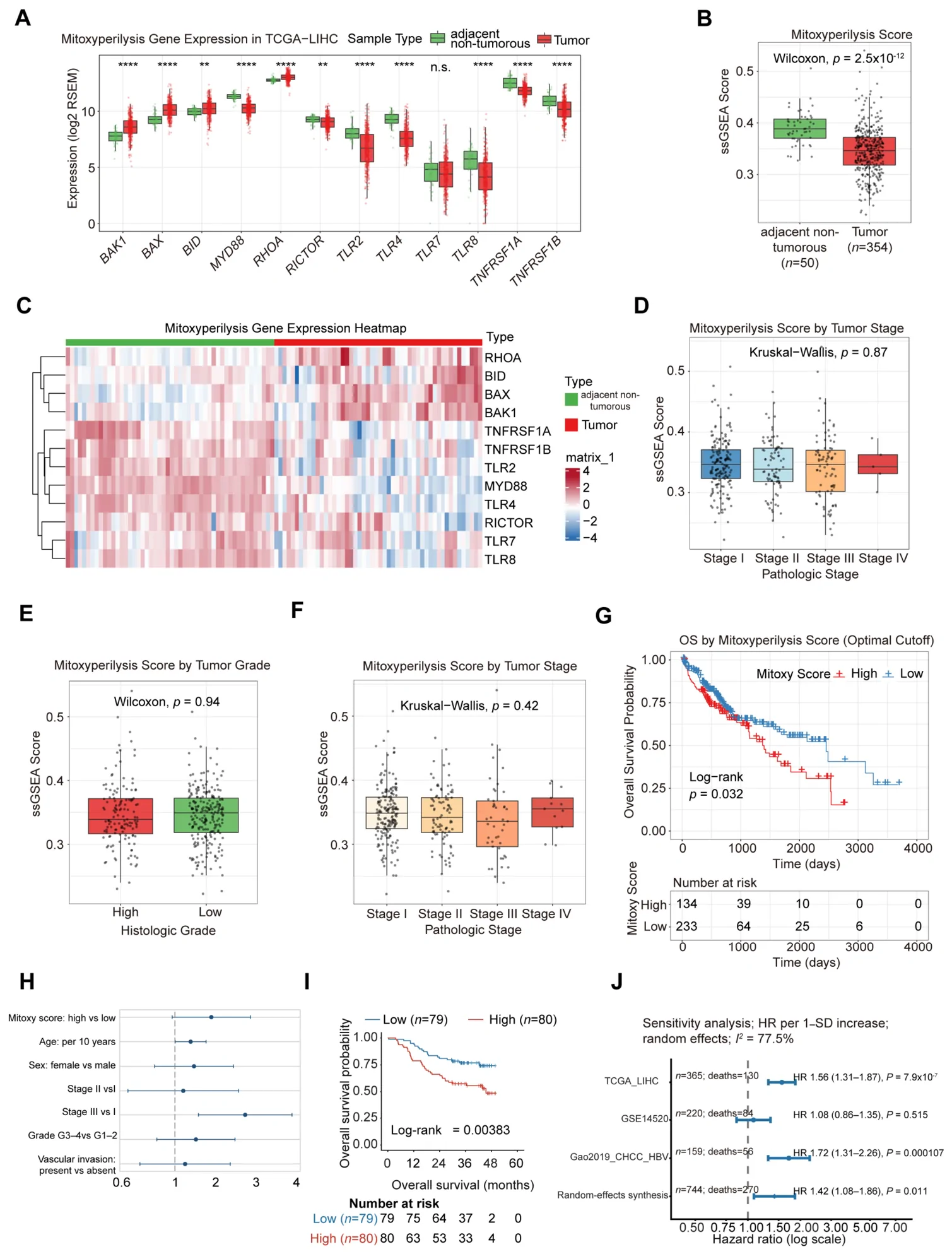
Expression and prognostic relevance of the mitoxyperilysis-related transcriptional program in hepatocellular carcinoma. (**A**) Expression of 12 mitoxyperilysis-related genes in TCGA-LIHC tumors (*n* = 354) and adjacent non-tumorous liver tissues (*n* = 50). (**B**) Comparison of ssGSEA-derived Mitoxyperilysis Scores between tumor and adjacent non-tumorous tissues (Wilcoxon rank-sum test, *p* = 2.5 × 10^−12^). (**C**) Heatmap showing the standardized expression patterns of the 12 genes across the two tissue groups. (**D**,**F**) Mitoxyperilysis Scores according to the available pathological-stage variables; neither comparison was significant (Kruskal–Wallis test, *p* = 0.87 and *p* = 0.42, respectively). (**E**) Comparison of Mitoxyperilysis Scores between high- and low-grade tumors (Wilcoxon rank-sum test, *p* = 0.94). (**G**) Kaplan–Meier overall survival curves for patients with high (*n* = 134) and low (*n* = 233) Mitoxyperilysis Scores in TCGA-LIHC (log-rank *p* = 0.032). (**H**) Multivariable Cox regression for overall survival, including the Mitoxyperilysis Score group, age per 10-year increase, sex, tumor stage, histological grade, and vascular invasion. Points indicate adjusted hazard ratios, horizontal lines indicate 95% confidence intervals, and the dashed vertical line denotes a hazard ratio of 1. (**I**) Independent Kaplan–Meier validation in the Gao2019 CHCC-HBV cohort (low, *n* = 79; high, *n* = 80; log-rank *p* = 0.00383). (**J**) Cohort-specific and pooled hazard ratios for overall survival per 1-standard-deviation increase in the continuous Mitoxyperilysis Score. The random-effects model included TCGA-LIHC (*n* = 365; 130 deaths), GSE14520 (*n* = 220; 84 deaths), and Gao2019 CHCC-HBV (*n* = 159; 56 deaths), yielding a pooled hazard ratio of 1.42 (95% CI, 1.08–1.86; *p* = 0.011; *I^2^* = 77.5%). Statistical significance in panel A is indicated as follows: ** *p* < 0.01; **** *p* < 0.0001; n.s., not significant.

The expression of most mitoxyperilysis-related genes differed significantly between 354 HCC tumors and 50 adjacent non-tumorous liver tissues in TCGA-LIHC (Figure 1A–C). The composite Mitoxyperilysis Score was lower in tumors than in adjacent non-tumorous tissues (*p* = 2.5 × 10^−12^; Figure 1B) but did not differ significantly according to pathological stage or histological grade (Figure 1D–F). Nevertheless, patients in the high-score group had shorter overall survival than those in the low-score group (log-rank *p* = 0.032; Figure 1G).

To determine whether this association was independent of established clinicopathological variables, we performed multivariable Cox regression incorporating age, sex, tumor stage, histological grade, and vascular invasion. The adjusted hazard ratio for the high-score group remained above 1, but its 95% confidence interval crossed 1, indicating that the score was not independently prognostic after adjustment. Advanced tumor stage, particularly stage III disease, remained the strongest adverse factor in the model (Figure 1H).

Because findings from a single cohort may be influenced by cohort-specific sampling and platform effects, survival analysis was extended to the independent Gao2019 CHCC-HBV cohort. High-score patients again showed significantly poorer overall survival (low, *n* = 79; high, *n* = 80; log-rank *p* = 0.00383; Figure 1I). In a sensitivity analysis treating the score as a continuous variable, higher scores were associated with increased mortality in TCGA-LIHC and Gao2019 CHCC-HBV, whereas the association in GSE14520 was weaker and non-significant. Random-effects integration of the three cohorts yielded a pooled hazard ratio of 1.42 per 1-standard-deviation increase in score (95% CI, 1.08–1.86; *p* = 0.011; Figure 1J). Although between-cohort heterogeneity was substantial (*I^2^* = 77.5%), the combined analysis supports a reproducible association between higher mitoxyperilysis-related transcriptional activity and unfavorable HCC survival.

### 3.2. Mitoxyperilysis Activity Stratifies Distinct Transcriptional Programs Enriched in Inflammatory and Immune Regulatory Pathways

**Figure 2:**
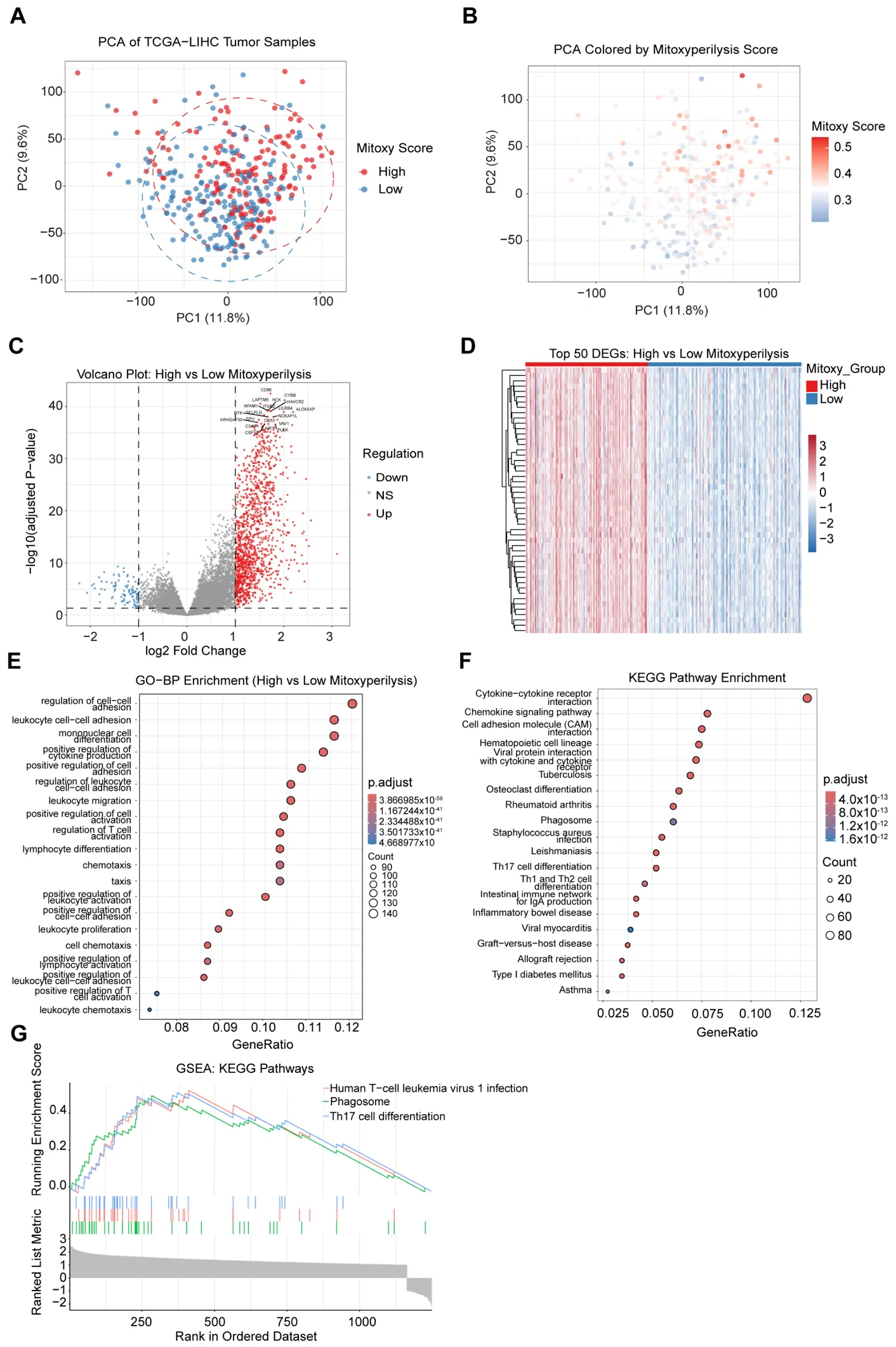
Transcriptome-wide differential analysis reveals inflammatory and immune regulatory gene programs as principal correlates of mitoxyperilysis activity. (**A**) Principal component analysis (PCA) scatter plot of TCGA-LIHC tumor transcriptomes, with individual specimens colored according to their binary mitoxyperilysis classification (red, high; blue, low). PC1 and PC2 explain 11.8% and 6%, respectively, of the total transcriptional variance. (**B**) PCA scatter plot identical in sample coordinates to panel (**A**), but with specimens colored according to a continuous gradient representing their numerical Mitoxyperilysis Score, ranging from low (blue) to high (red/orange). This representation illustrates the graded transcriptional divergence associated with incremental changes in mitoxyperilysis activity. (**C**) Volcano plot depicting genome-wide differential expression between high- and low-mitoxyperilysis tumor subgroups in TCGA-LIHC. Each point represents a single gene; the horizontal dashed line indicates the threshold for statistical significance (adjusted *p* < 0.05), and vertical dashed lines demarcate the log_2_ fold change boundaries (|log_2_FC| > 1). Upregulated genes are shown in red, downregulated genes in blue, and non-significant genes in gray. (**D**) Heatmap of z-score normalized expression values for the top 50 differentially expressed genes (DEGs) ranked by adjusted *p*-value, displayed across high- (red) and low- (blue) mitoxyperilysis patient subgroups. Hierarchical clustering was applied to both rows and columns to reveal co-expression structures associated with each mitoxyperilysis phenotype. (**E**) Bubble plot of Gene Ontology Biological Process (GO-BP) terms significantly enriched among DEGs between high- and low-mitoxyperilysis groups. Bubble size reflects the number of DEGs annotated to each term, while color gradient represents the Benjamini–Hochberg adjusted *p*-value. Displayed terms are ranked by gene ratio and include immune cell biology, leukocyte migration, and chemotaxis-related processes. (**F**) Bubble plot of KEGG pathway enrichment analysis performed on the same DEG set as panel E. Terms are ordered by gene ratio along the *x*-axis; bubble size encodes gene count and color intensity reflects adjusted *p*-value magnitude. Pathways spanning cytokine–cytokine receptor interaction, chemokine signaling, and lymphocyte differentiation dominate the enrichment landscape. (**G**) Gene Set Enrichment Analysis (GSEA) running enrichment score plots for three representative KEGG gene sets—Human T-cell leukemia virus 1 infection, Phagosome, and Th17 cell differentiation—demonstrating positive enrichment in the high-mitoxyperilysis tumor phenotype. Vertical tick marks at the bottom of each panel indicate the positions of gene set members within the ranked gene list.

Having established the prognostic relevance of the Mitoxyperilysis Score, we sought to characterize the transcriptional landscape that distinguishes tumors with divergent mitoxyperilysis activity. Principal component analysis of the TCGA-LIHC tumor transcriptome demonstrated a discernible spatial separation between high- and low-mitoxyperilysis specimens along the primary axes of variation (Figure 2A,B), indicating that mitoxyperilysis activity is associated with broad and coordinated transcriptomic differences rather than isolated gene expression changes. Differential expression analysis between the two subgroups identified a substantial number of DEGs, which were visualized in a volcano plot demonstrating a preponderance of upregulated transcripts in the high-mitoxyperilysis tumor subclass (Figure 2C). The top-ranked differentially expressed genes exhibited a highly ordered expression pattern in the heatmap, with high-mitoxyperilysis specimens clustered together and characterized by a distinct transcriptional signature relative to their low-mitoxyperilysis counterparts (Figure 2D).

Functional annotation of the DEG set through GO biological process enrichment analysis revealed a predominant overrepresentation of terms directly related to leukocyte biology and inflammatory signaling, including leukocyte cell–cell adhesion, leukocyte migration, regulation of T cell activation, lymphocyte differentiation, chemotaxis, and cell chemotaxis, all achieving statistical significance after multiple testing correction (adjusted *p*-values ranging from ~3.87 × 10^−59^ to ~4.67 × 10^−41^; Figure 2E). Parallel KEGG pathway enrichment analysis converged on analogous immunological themes, with the cytokine–cytokine receptor interaction, chemokine signaling pathway, cell adhesion molecule (CAM) interaction, hematopoietic cell lineage, and Th17 cell differentiation pathways among the most significantly enriched terms (Figure 2F). Complementing these ORA findings, GSEA corroborated the activation of immune-related gene sets in high-mitoxyperilysis tumors, with pathways including Human T-cell leukemia virus 1 infection, Phagosome, and Th17 cell differentiation displaying positive enrichment scores in the high-mitoxyperilysis phenotype (Figure 2G). Taken together, these multi-method enrichment analyses converge on a coherent interpretation: high mitoxyperilysis activity in HCC is coupled with transcriptional programs that are fundamentally oriented toward immune regulation and inflammatory signaling, prompting our subsequent inquiry into the tumor immune microenvironment.

### 3.3. Elevated Mitoxyperilysis Activity Associates with Enhanced Microenvironmental Infiltration and Upregulation of Immune Checkpoint Molecules

**Figure 3:**
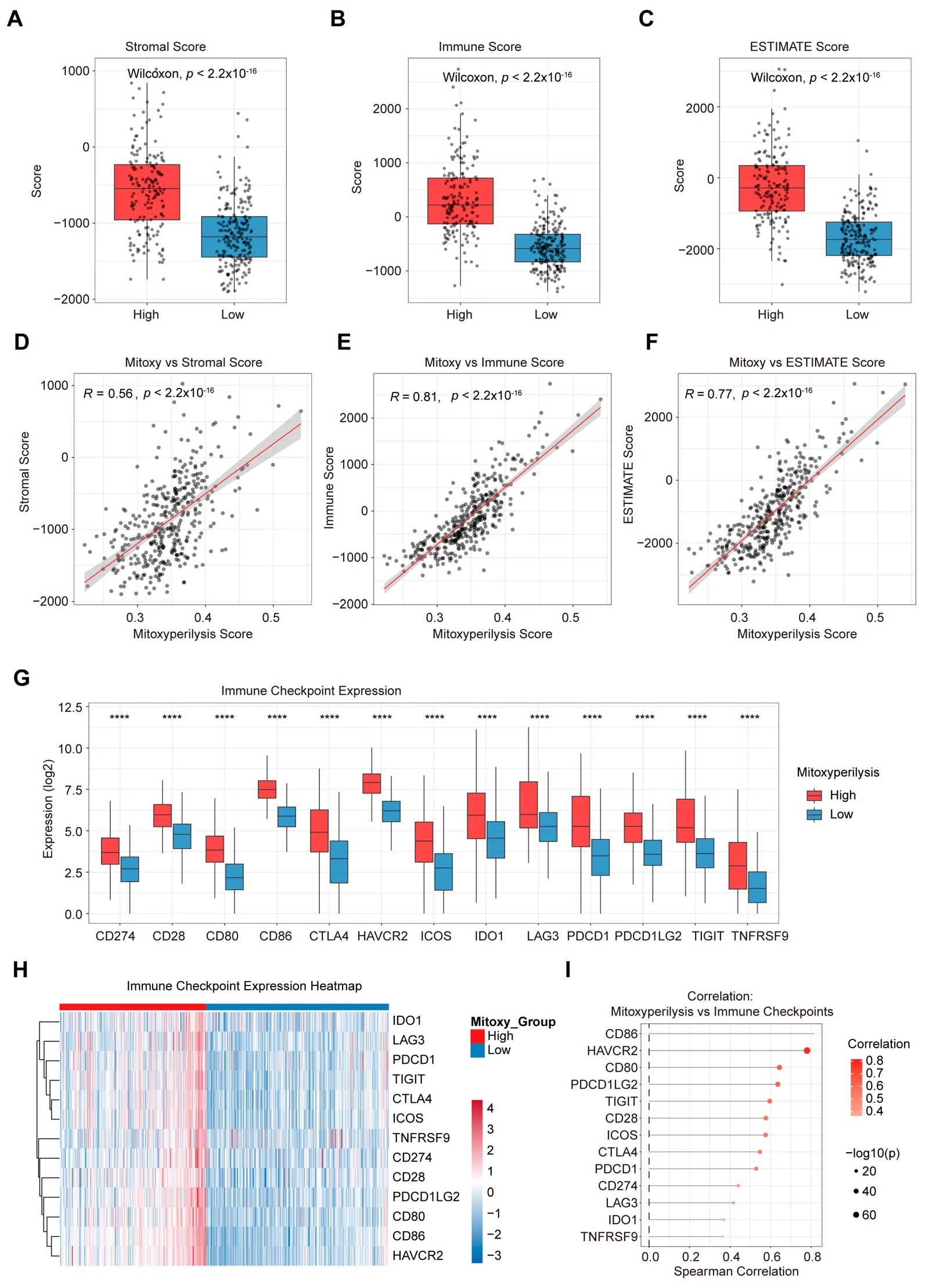
Elevated mitoxyperilysis activity delineates an immunologically enriched tumor microenvironment with coordinate upregulation of immune checkpoint molecules. (**A**–**C**) Box plots comparing ESTIMATE algorithm-derived (**A**) Stromal Score, (**B**) Immune Score, and (**C**) ESTIMATE Score between high- and low-mitoxyperilysis tumor subgroups in TCGA-LIHC. All three scores were significantly elevated in the high-mitoxyperilysis group (Wilcoxon rank-sum test, all *p* < 2.2 × 10^−16^), indicating enhanced stromal and immune cellular infiltration associated with elevated mitoxyperilysis activity. (**D**–**F**) Scatter plots with fitted linear regression lines (red) and 95% confidence intervals (shaded regions) illustrating Spearman correlations between the continuous Mitoxyperilysis Score and (**D**) Stromal Score (R = 0.56, *p* < 2.2 × 10^−16^), (**E**) Immune Score (R = 0.81, *p* < 2.2 × 10^−16^), and (**F**) ESTIMATE Score (R = 0.77, *p* < 2.2 × 10^−16^) across TCGA-LIHC tumor samples. Each data point corresponds to an individual tumor specimen. (**G**) Grouped box plots depicting expression levels (log_2_ transformed) of twelve immune checkpoint and co-stimulatory molecules—*CD274*, *CD28*, *CD80*, *CD86*, *CTLA4*, *HAVCR2*, *ICOS*, *IDO1*, *LAG3*, *PDCD1*, *PDCD1LG2*, and *TIGIT*—stratified by mitoxyperilysis group (red, high; blue, low) in TCGA-LIHC. All molecules were significantly upregulated in the high-mitoxyperilysis subgroup (**** *p* < 0.0001 by Wilcoxon rank-sum test with multiple testing correction). (**H**) Hierarchically clustered heatmap displaying z-score normalized expression of all twelve immune checkpoint molecules across individual TCGA-LIHC tumor specimens, annotated by mitoxyperilysis group designation. Clustering reveals a coherent pattern of coordinate checkpoint upregulation in the high-mitoxyperilysis subtype. (**I**) Dot matrix overview summarizing Spearman correlation coefficients between the Mitoxyperilysis Score and each immune checkpoint molecule across the full TCGA-LIHC cohort. Dot size reflects the −log_10_ (*p*-value) and color encodes correlation magnitude, demonstrating uniformly positive and statistically robust associations for all evaluated molecules.

Guided by the immunological enrichment signatures identified above, we next applied the ESTIMATE algorithm to quantify the cellular composition of the tumor microenvironment across the TCGA-LIHC cohort. The high-mitoxyperilysis patient subgroup demonstrated markedly elevated Stromal Scores (Wilcoxon, *p* < 2.2 × 10^−16^; Figure 3A), Immune Scores (Wilcoxon, *p* < 2.2 × 10^−16^; Figure 3B), and composite ESTIMATE Scores (Wilcoxon, *p* < 2.2 × 10^−16^; Figure 3C) relative to their low-mitoxyperilysis counterparts, indicating a substantially heightened non-malignant cellular infiltrate in tumors with active mitoxyperilysis. These group-level differences were reinforced by continuous correlation analyses, which revealed that the Mitoxyperilysis Score maintained significant positive correlations with each of the three ESTIMATE metrics: Stromal Score (R = 0.56, *p* < 2.2 × 10^−16^; Figure 3D), Immune Score (R = 0.81, *p* < 2.2 × 10^−16^; Figure 3E), and ESTIMATE Score (R = 0.77, *p* < 2.2 × 10^−16^; Figure 3F). The particularly robust correlation with the Immune Score suggests that immune cell infiltration, rather than stromal remodeling alone, constitutes the principal microenvironmental correlate of mitoxyperilysis activity.

Given this strong immune infiltration signature, we subsequently characterized the expression of canonical immune regulatory molecules across mitoxyperilysis subgroups. All twelve interrogated immune checkpoint and co-stimulatory molecules—including *CD274, CD28, CD80, CD86, CTLA4, HAVCR2, ICOS, IDO1, LAG3, PDCD1, PDCD1LG2*, and *TIGIT*—were significantly upregulated in the high-mitoxyperilysis group relative to the low-mitoxyperilysis group (all *p* < 0.0001; Figure 3G). Hierarchical clustering of checkpoint molecule expression across individual samples reinforced this finding, revealing a coherent co-upregulation pattern in high-mitoxyperilysis specimens (Figure 3H). Spearman correlation analysis further demonstrated that the Mitoxyperilysis Score maintained consistently positive and statistically significant associations with each of these immune molecules, with the strongest correlations observed for CD86, HAVCR2, and CD80 (Figure 3I). These data collectively indicate that elevated mitoxyperilysis activity defines an HCC subtype characterized by a highly infiltrated yet immunosuppression-prone microenvironment, as reflected by the concurrent upregulation of both stimulatory and inhibitory immune regulatory checkpoints.

### 3.4. M2-Polarized Macrophages Are the Dominant Immune Cell Population Consistently Associated with Elevated Mitoxyperilysis Activity, and Mitoxyperilysis-Induced Hepatoma Cells Promote Macrophage M2 Skewing Ex Vivo

**Figure 4:**
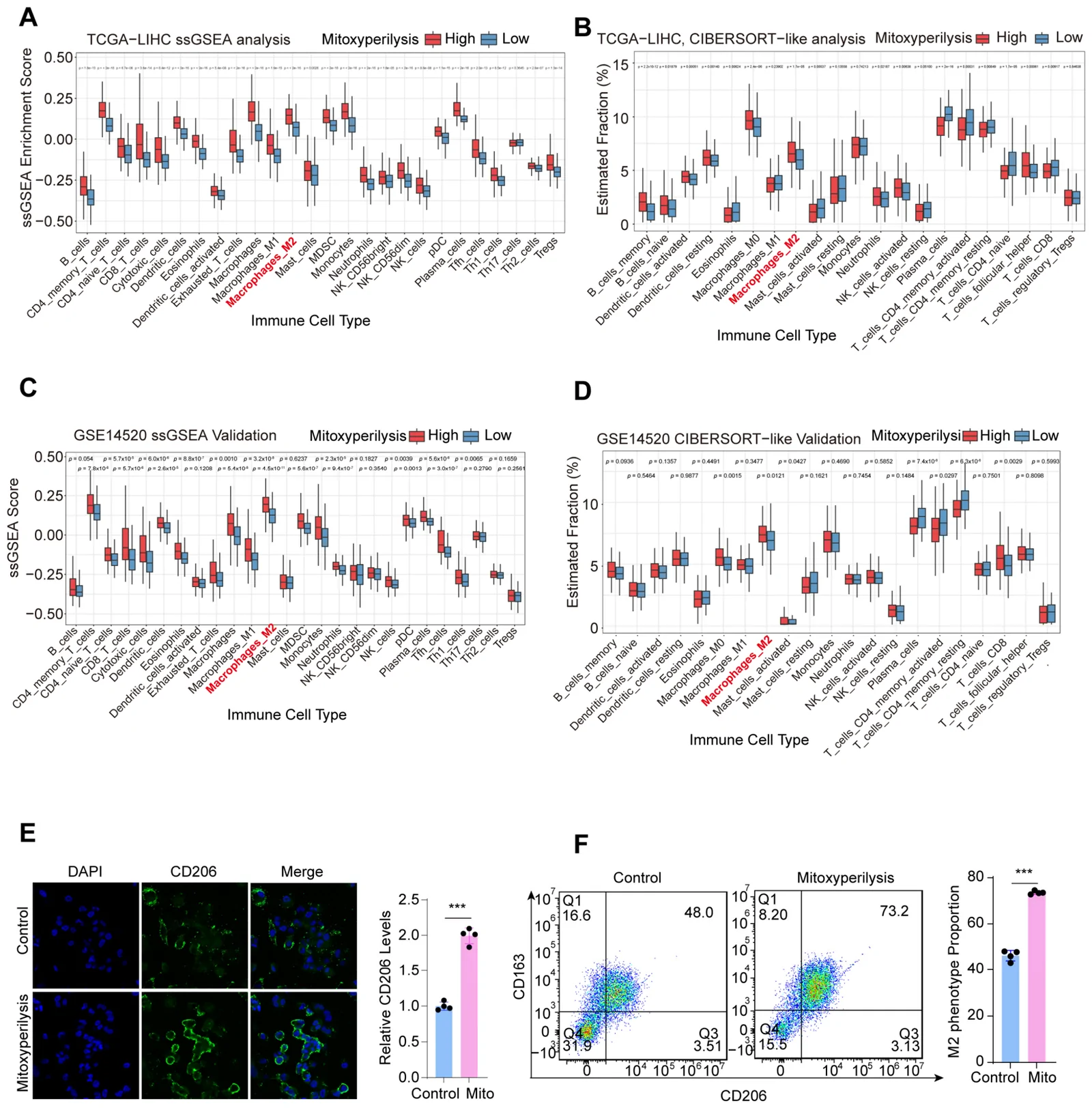
M2-polarized macrophages are consistently enriched in high-mitoxyperilysis hepatocellular carcinoma, and mitoxyperilysis induction experimentally drives macrophage M2 polarization. (**A**) ssGSEA-based enrichment scores for 23 immune cell populations, compared between high- (red) and low- (blue) mitoxyperilysis patient subgroups in the TCGA-LIHC cohort. M2 macrophages (highlighted in bold red text) exhibit one of the most pronounced and significant elevations in the high-mitoxyperilysis group relative to all other evaluated immune subsets. (**B**) CIBERSORT-like deconvolution-estimated fractional compositions of 22 immune cell subpopulations in TCGA-LIHC specimens, stratified by mitoxyperilysis group. Estimated M2 macrophage fractions (highlighted) are significantly elevated in the high-mitoxyperilysis subgroup, consistent with ssGSEA findings in panel A. (**C**) Validation of ssGSEA-derived immune cell infiltration profiles in the independent GSE14520 cohort, with annotated *p*-values for pairwise comparisons between mitoxyperilysis subgroups. M2 macrophage enrichment scores are significantly higher in the high-mitoxyperilysis group, reproducing the observation from the discovery cohort. (**D**) Independent validation of CIBERSORT-like immune deconvolution results in GSE14520. M2 macrophage estimated fractions remain significantly elevated in the high-mitoxyperilysis subgroup, confirming cross-platform concordance of this immune cell association. (**E**) Representative immunofluorescence images of macrophages co-cultured with control or mitoxyperilysis-induced hepatoma cells, stained with DAPI (blue, nuclei) and anti-CD206 antibody (green, M2 macrophage marker). Scale bars are indicated; *n* = 4 for each group. The quantification bar graph on the right displays relative CD206 fluorescence intensity normalized to the control condition, demonstrating a marked increase in CD206 protein levels upon co-culture with mitoxyperilysis-induced tumor cells. (**F**) Flow cytometric analysis of macrophage polarization using dual-surface staining for CD163 and CD206, which together define the M2 macrophage phenotype. Representative bivariate dot plots are shown for control and mitoxyperilysis co-culture conditions, with the percentage of CD163^+^CD206^+^ double-positive cells indicated in the upper-right quadrant (Control: 48.0%; Mitoxyperilysis: 73.2%). The bar graph on the right summarizes the M2 macrophage proportion across biological replicates; *n* = 4 for each group.

To delineate the specific immune cell populations whose infiltration is coupled with mitoxyperilysis activity, we applied two orthogonal computational deconvolution strategies—ssGSEA-based immune profiling and CIBERSORT-like fractional estimation—to both the TCGA-LIHC discovery cohort and the GSE14520 validation cohort. In TCGA-LIHC, ssGSEA-based analysis revealed differential enrichment across multiple immune cell subsets between high- and low-mitoxyperilysis tumors; among these, M2-polarized macrophages emerged as one of the most prominently and significantly elevated populations in the high-mitoxyperilysis group (Figure 4A). CIBERSORT-like deconvolution applied to the same cohort corroborated this observation, with estimated M2 macrophage fractions significantly higher in high-mitoxyperilysis specimens compared to those with low activity (Figure 4B). Critically, both analytic approaches yielded concordant results upon validation in the independent GSE14520 dataset, where M2 macrophages again stood out as a consistently and significantly elevated cell type in high-mitoxyperilysis tumors across both ssGSEA (Figure 4C) and CIBERSORT-like analyses (Figure 4D), while the directional changes in other immune cell populations were less consistently reproduced between cohorts.

To determine whether this association reflects a causal relationship rather than mere co-occurrence, we established an indirect co-culture system in which PMA-differentiated macrophages were exposed to conditioned signals derived from HCC cells in which mitoxyperilysis had been experimentally induced. Immunofluorescence staining for CD206, a canonical mannose receptor marking M2-polarized macrophages, demonstrated a pronounced elevation in CD206 fluorescence intensity in macrophages co-cultured with mitoxyperilysis-induced hepatoma cells relative to control conditions (Figure 4E). This finding was independently confirmed by flow cytometric quantification using dual-marker surface staining: the proportion of CD163^+^CD206^+^ double-positive cells—operationally defining the M2 macrophage phenotype—increased substantially from approximately 48.0% in the control condition to 73.2% in the mitoxyperilysis co-culture setting (Figure 4F). These experimental results provide direct mechanistic evidence that mitoxyperilysis induction in hepatoma cells confers upon them the capacity to actively redirect macrophage phenotypic identity toward an M2 immunosuppressive state through paracrine mechanisms.

### 3.5. Single-Cell Intercellular Communication Analysis Identifies ANXA1-FPR Signaling as the Principal Communication Axis Between Mitoxyperilysis-High Cancer Cells and Myeloid Cells

**Figure 5:**
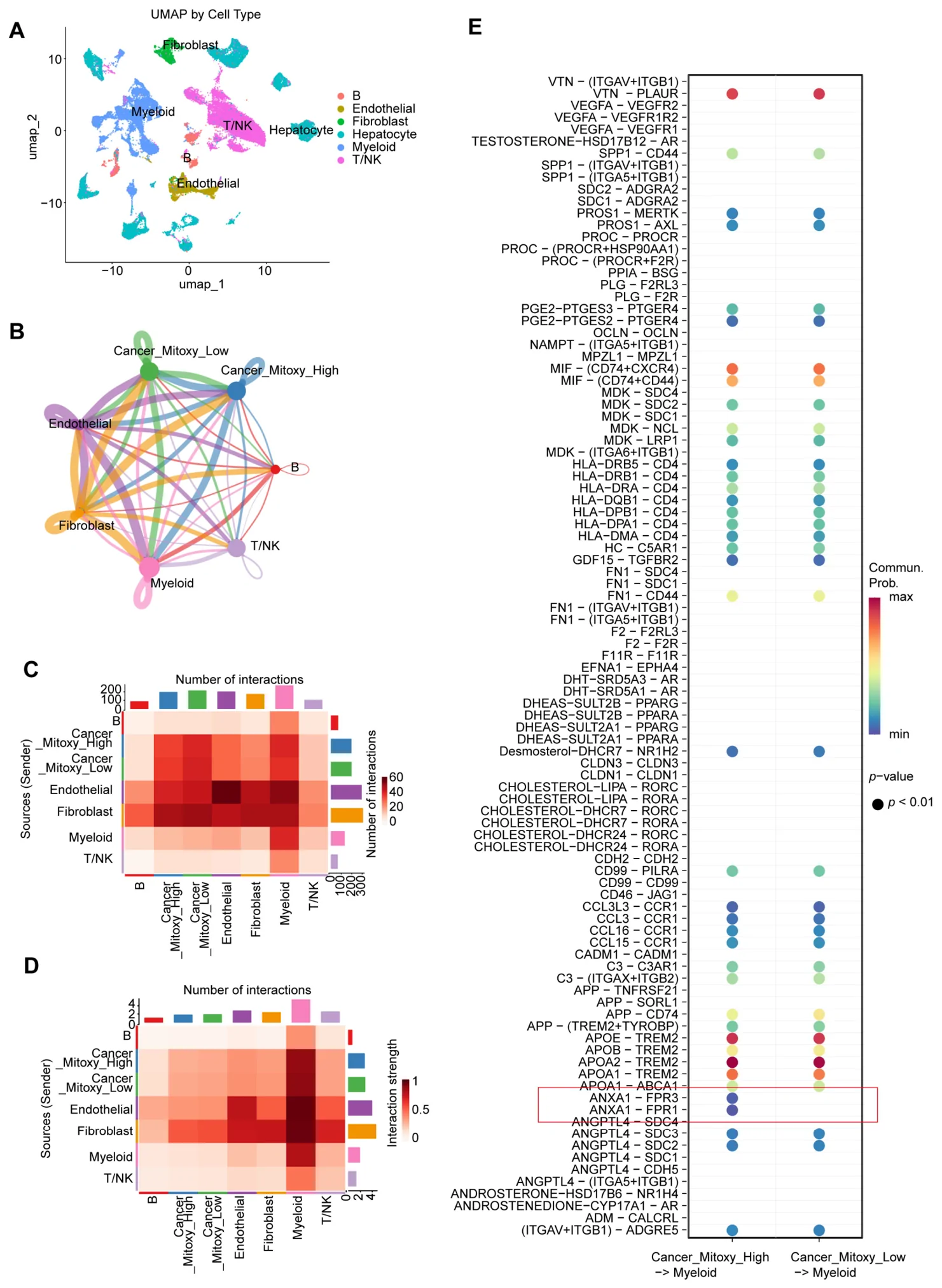
Single-cell resolution intercellular communication analysis identifies ANXA1–FPR signaling as the dominant axis linking mitoxyperilysis-active cancer cells to myeloid cell reprogramming. (**A**) UMAP visualization of the reanalyzed GSE149614 scRNA-seq dataset, which originally comprised 71,915 cells from 21 specimens obtained from 10 patients with HCC, including primary tumors, non-tumor liver tissues, portal vein tumor thrombi, and a metastatic lymph node: B cells (dark blue), endothelial cells (yellow), fibroblasts (green), hepatocytes (olive), myeloid cells (pink), and T/NK cells (teal). Major cell lineages are indicated by distinct color codes. (**B**) Chord diagram depicting the global intercellular communication network among all major cell populations, with hepatocyte clusters further resolved into Cancer_Mitoxy_High and Cancer_Mitoxy_Low subpopulations based on per-cell mitoxyperilysis ssGSEA scores. Edge thickness and color are proportional to the aggregate communication probability between indicated cell type pairs, highlighting qualitative differences in the communicative topology of the two cancer subpopulations. (**C**) Heatmap quantifying the number of inferred ligand–receptor interactions from each sender cell population (rows) to each receiver population (columns), with color intensity reflecting interaction count. Mitoxyperilysis-high cancer cells display substantially greater interaction numbers directed toward the myeloid compartment compared to their mitoxyperilysis-low counterparts. (**D**) Heatmap summarizing overall intercellular communication strength (weighted interaction probability) from each sender to each receiver cell type. The Cancer_Mitoxy_High subpopulation demonstrates heightened interaction strength with myeloid cells relative to the Cancer_Mitoxy_Low subpopulation, consistent with their amplified paracrine signaling capacity. (**E**) Dot plot comparing specific ligand–receptor pair communication probabilities in the Cancer_Mitoxy_High → Myeloid versus Cancer_Mitoxy_Low → Myeloid communication axes. Dot size encodes statistical significance (filled dots indicate *p* < 0.01), and color gradient reflects communication probability from minimum (blue) to maximum (red). The red-bordered region highlights ANXA1–FPR3 and ANXA1–FPR1 interactions, which are selectively and substantially enriched in the high-mitoxyperilysis cancer cell → myeloid communication axis and absent or negligible in the low-mitoxyperilysis counterpart.

To achieve single-cell resolution of the intercellular communication mechanisms underlying macrophage polarization by mitoxyperilysis-active tumor cells, we leveraged a publicly available HCC single-cell RNA sequencing dataset. Following standard quality control filtering and dimensionality reduction, major cell lineages—including B cells, endothelial cells, fibroblasts, hepatocytes, myeloid cells, and T/NK cells—were resolved by UMAP visualization (Figure 5A). Hepatocyte clusters were further stratified into Cancer_Mitoxy_High and Cancer_Mitoxy_Low subpopulations based on per-cell ssGSEA mitoxyperilysis scoring, enabling a direct comparison of their communicative landscapes with the myeloid compartment.

Chord diagram visualization of global intercellular interaction networks demonstrated that the Cancer_Mitoxy_High subpopulation engaged in qualitatively and quantitatively distinct communication patterns relative to Cancer_Mitoxy_Low cells, with particularly notable differences in interactions directed toward myeloid cells (Figure 5B). Quantitative assessment of interaction numbers (Figure 5C) and interaction strengths (Figure 5D) confirmed that high-mitoxyperilysis cancer cells exhibited amplified communicative activity toward the myeloid compartment, consistent with their putative role in driving macrophage phenotypic reprogramming. Systematic comparison of specific ligand–receptor pairs active in the Cancer_Mitoxy_High → Myeloid versus Cancer_Mitoxy_Low → Myeloid communication axes revealed a diverse landscape of differential interactions; however, the *ANXA1-FPR3* and *ANXA1-FPR1* ligand–receptor pairs within the ANXA1–FPR signaling module were specifically and selectively enriched in the high-mitoxyperilysis cancer cell → myeloid communication axis (Figure 5E, highlighted region), with communication probabilities substantially exceeding those observed in the low-mitoxyperilysis condition. These findings pinpoint ANXA1-mediated formyl peptide receptor engagement as a candidate paracrine mechanism by which mitoxyperilysis-active tumor cells orchestrate myeloid cell reprogramming.

### 3.6. ANXA1 Links Mitoxyperilysis-Related Tumor-Cell Stress to M2-like Macrophage Polarization

**Figure 6:**
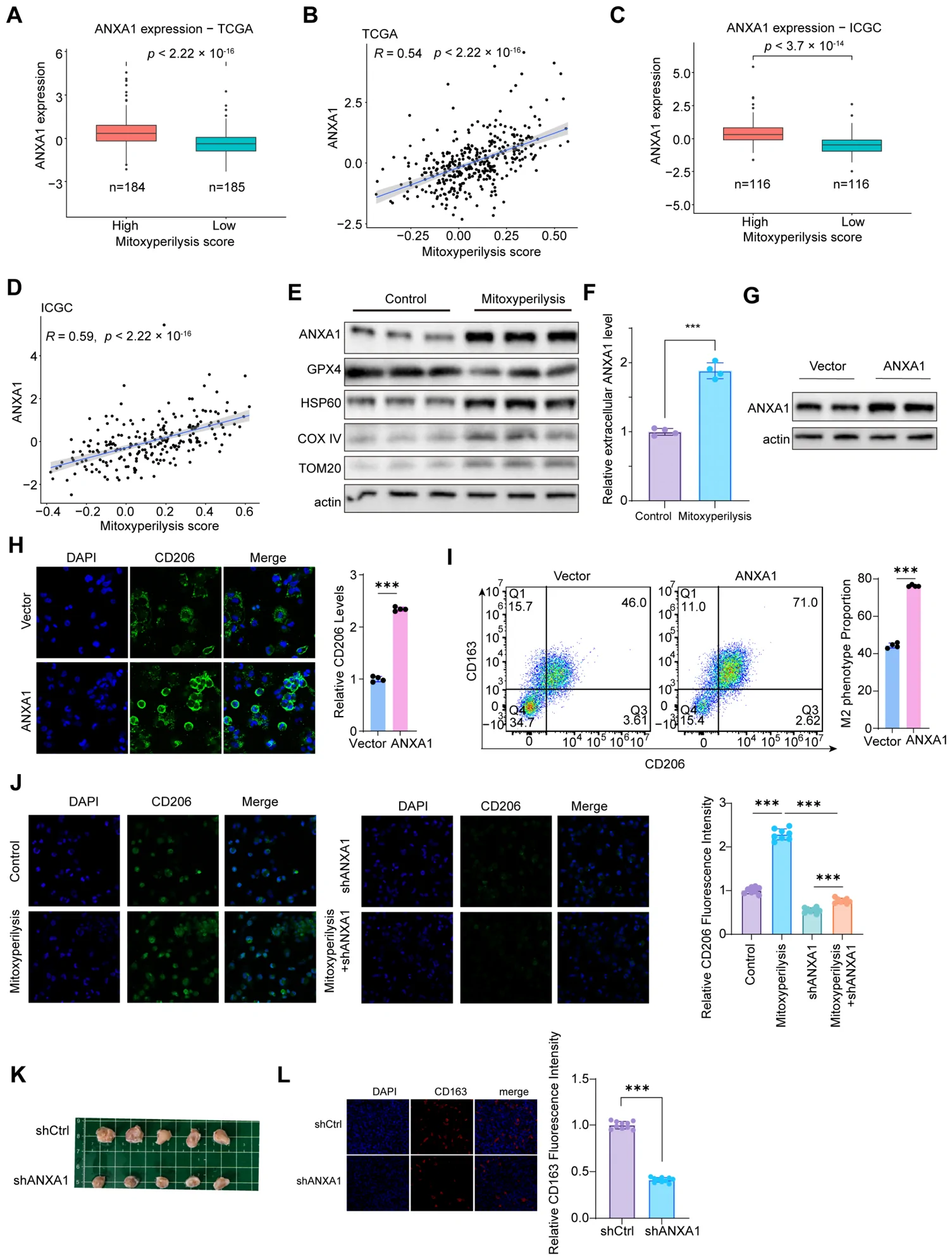
Mitoxyperilysis promotes extracellular ANXA1 release and ANXA1-dependent M2-like macrophage polarization. (**A**) ANXA1 expression in high- (*n* = 184) and low-score (*n* = 185) TCGA-LIHC tumors (Wilcoxon rank-sum test, *p* < 2.22 × 10^−16^). (**B**) Correlation between *ANXA1* expression and the continuous Mitoxyperilysis Score in TCGA-LIHC (Spearman *R* = 0.54, *p* < 2.2 × 10^−16^). (**C**) ANXA1 expression in high- and low-score tumors from the ICGC cohort (*n* = 116 per group; *p* = 3.7 × 10^−14^). (**D**) Correlation between *ANXA1* expression and the Mitoxyperilysis Score in ICGC (Spearman *R* = 0.59, *p* < 2.2 × 10^−16^) In panels B and D, the blue line represents the fitted linear regression line, and the shaded region indicates the 95% confidence interval. (**E**) Western blot analysis of ANXA1, GPX4, HSP60, COX IV, and TOM20 in control and mitoxyperilysis-induced Huh-7 cells. Lanes 1–3 represent independent control samples, and lanes 4–6 represent independent mitoxyperilysis-induced samples. β-Actin served as the loading control. The mitochondrial proteins were assessed as supportive biochemical measurements and were not considered specific markers of mitoxyperilysis. (**F**) ELISA measurement of extracellular ANXA1 in conditioned media from control and mitoxyperilysis-induced Huh-7 cells. Values were normalized to the control mean; each point represents an independent biological replicate. (**G**) Western blot confirmation of ANXA1 overexpression. Lanes 1–2 represent empty-vector controls, and lanes 3–4 represent ANXA1-overexpressing Huh-7 cells. (**H**) Representative immunofluorescence images and quantification of CD206 expression in macrophages co-cultured with vector-control or ANXA1-overexpressing Huh-7 cells. DAPI, blue; CD206, green; *n* = 4 for each group. (**I**) Representative flow-cytometric plots and quantification of CD163^+^CD206^+^ M2-like macrophages following co-culture with vector-control or ANXA1-overexpressing Huh-7 cells; *n* = 4 for each group. (**J**) Representative CD206 immunofluorescence images and quantification from the co-culture loss-of-function experiment. Mitoxyperilysis induction increased macrophage CD206 expression, whereas ANXA1 knockdown substantially reduced this response. The four groups were control, mitoxyperilysis, shANXA1, and mitoxyperilysis plus shANXA1; *n* = 8 for each group. (**K**) Representative images of subcutaneous tumors generated using shCtrl- or shANXA1-transduced HCC cells (*n* = 5 per group). (**L**) Representative tumor-tissue immunofluorescence images and quantification of relative CD163 fluorescence intensity. ANXA1 knockdown significantly reduced intratumoral CD163 staining, consistent with decreased M2-like macrophage infiltration. DAPI, blue; CD163, red. Data are presented as mean ± SD where applicable; *n* = 5 for each group; *** *p* < 0.001.

*ANXA1* expression was significantly higher in high-mitoxyperilysis tumors in both TCGA-LIHC and ICGC (Figure 6A,C). It also correlated positively with the continuous Mitoxyperilysis Score in TCGA-LIHC (*R* = 0.54, *p* < 2.2 × 10^−16^) and ICGC (*R* = 0.59, *p* < 2.2 × 10^−16^; Figure 6B,D), demonstrating that this association was reproducible across independent cohorts.

Mitoxyperilysis induction increased intracellular ANXA1 protein and was accompanied by changes in mitochondria-associated proteins used as supportive biochemical measurements (Figure 6E). ELISA further showed an approximately 1.9-fold increase in extracellular ANXA1 in conditioned medium from mitoxyperilysis-induced Huh-7 cells (Figure 6F), supporting the availability of tumor-derived ANXA1 for paracrine signaling. ANXA1 overexpression increased macrophage CD206 expression and raised the CD163^+^CD206^+^ M2-like fraction from approximately 46% to 71% (Figure 6G–I). The original uncropped Western blot images corresponding to Figure 6E,G are provided in Figure S1.

Loss-of-function experiments further demonstrated that ANXA1 contributes to the macrophage-polarizing effect of mitoxyperilysis. Although mitoxyperilysis-induced Huh-7 cells markedly increased macrophage CD206 expression, ANXA1 knockdown substantially attenuated this response in the co-culture system (Figure 6J). The incomplete suppression observed after ANXA1 knockdown suggests that ANXA1 is an important but not exclusive mediator. Consistent with the in vitro findings, ANXA1 knockdown reduced subcutaneous tumor burden in vivo (Figure 6K). Tumor-tissue immunofluorescence further showed a significant reduction in CD163 staining after ANXA1 knockdown, indicating decreased infiltration or accumulation of M2-like tumor-associated macrophages (Figure 6L). Together, these findings support an ANXA1-dependent link between mitoxyperilysis-related tumor-cell stress, macrophage M2 polarization, and HCC progression.

## 4. Discussion

The present study provides an integrated characterization of mitoxyperilysis-related transcriptional features in HCC. Tumors with relatively higher Mitoxyperilysis-Related Transcriptional Scores exhibited poorer overall survival, increased immune and stromal infiltration, enhanced immune-checkpoint expression, and enrichment of M2-like macrophages. CellChat analysis and in vitro experiments further suggested that ANXA1–FPR signaling may represent a potential link between mitoxyperilysis-related tumor-cell stress and macrophage M2 polarization [26,27].

The lower mean Mitoxyperilysis-Related Transcriptional Score observed in HCC tissues compared with non-tumor liver and the poorer prognosis of the relatively high-score tumor subgroup are not necessarily contradictory. The former reflects an overall difference between malignant and non-malignant tissues, whereas the latter reflects residual transcriptional heterogeneity among tumors. Thus, although the mitoxyperilysis-related program is globally attenuated in HCC, tumors retaining relatively higher expression of this program may exhibit distinct biological and clinical characteristics. Importantly, the score was strongly correlated with the ESTIMATE Immune Score and was accompanied by increased immune-checkpoint expression and M2-like macrophage enrichment. Therefore, its prognostic association may partly reflect an immune-enriched but functionally immunosuppressive or exhausted tumor microenvironment, rather than tumor-cell-intrinsic mitoxyperilysis alone. Accordingly, the score should be interpreted as a mechanism-informed transcriptomic proxy, and the potential contribution of immune-cell composition cannot be excluded.

In recent years, the role of ANXA1 in regulating the immune microenvironment of HCC has attracted increasing attention. Studies have shown that ANXA1 is significantly overexpressed in HCC stromal cells (particularly macrophages), and its expression levels are positively correlated with programmed death-ligand 1 (PD-L1), suggesting its involvement in the establishment of an immunosuppressive microenvironment [26]. Mechanistically, ANXA1 drives the polarization of tumor-associated macrophages (TAMs) toward the M2 phenotype by activating the PI3K/AKT signaling pathway [26]. These M2-polarized macrophages secrete factors such as TGF-β, which further promote the activation of cancer-associated fibroblasts and the suppression of CD8^+^ T-cell function, thereby establishing an immunosuppressive tumor microenvironment (TME) [16]. In vitro and in vivo experiments confirm that human recombinant ANXA1 (hrANXA1) enhances TAM infiltration and M2 polarization, accelerating HCC progression and metastasis; conversely, ANXA1 knockdown reverses the M1/M2 ratio, inhibits tumor growth, and enhances T-cell activation [27]. Furthermore, tumor-derived ANXA1 is delivered via extracellular vesicles and regulates the polarization fate of macrophages; its absence significantly impairs the acquisition of the M2 phenotype [28]. Clinical data further indicate that high ANXA1 expression is closely associated with poor prognosis in HCC patients, and myeloid-specific ANXA1 deficiency can inhibit tumor progression. In summary, ANXA1 induces M2 polarization of TAMs through multiple pathways, playing a critical role in HCC immune evasion and malignant progression, and holds promise as a novel target for HCC immunotherapy [20].

Although lytic cell death is often considered pro-inflammatory, its effects on the tumor microenvironment are context dependent. Tumor cells exposed to mitoxyperilysis-inducing conditions may release or upregulate immunoregulatory mediators that promote tissue-repair-like macrophage responses. In the present study, ANXA1 expression increased under the mitoxyperilysis-inducing condition, and ANXA1 overexpression was sufficient to promote an M2-like macrophage phenotype. Together with the consistent enrichment of estimated M2 macrophages in high-score tumors, these findings suggest that mitoxyperilysis-related tumor-cell stress may contribute to an immunosuppressive microenvironment through ANXA1-associated macrophage polarization.

Recent studies have revealed a novel form of lytic cell death—mitoxyperilysis—and significant progress has been made in understanding its association with immune regulation. Wang et al. found that this process is synergistically triggered by innate immune agonists and metabolic stress (such as nutrient deprivation), leading to sustained contact between mitochondria and the plasma membrane, which induces localized oxidative damage and membrane rupture. This process is finely regulated by the mTORC2 pathway; in vivo activation of this pathway can induce tumor regression via mTORC2, suggesting its potential in reshaping the anti-tumor immune microenvironment [16]. Notably, mitoxyperilysis operates independently of classical death pathways (such as pyroptosis and necrotic apoptosis), driving membrane lysis through the integration of immune and metabolic signals, thereby providing new targets for immunotherapeutic interventions in sepsis and cancer. Expanding further into the broader framework of regulated cell death (RCD), multiple RCD modes are deeply involved in immune regulation: immunogenic cell death (ICD) activates adaptive immune responses by releasing damage-associated molecular patterns (DAMPs) [11]; PANoptosis integrates pyroptosis, apoptosis, and necroptosis to amplify inflammatory signaling [27]; meanwhile, lytic cell death pathways such as ferroptosis and necroptosis activate pathways like cGAS-STING and TLR9 through the release of mitochondrial DNA (mtDNA), thereby regulating macrophage polarization and T-cell function [29]. As a central hub, mitochondria not only mediate various forms of RCD (such as apoptosis and ferroptosis) but also finely regulate immune cell activation and the tumor microenvironment through metabolic reprogramming, dynamic remodeling, and mtROS signaling [20,30]. These mechanisms collectively highlight the dynamic interaction between RCD and immune networks, laying the theoretical foundation for precision therapies targeting the mitochondria-immune axis.

Mitoxyperilysis should be considered alongside, but not conflated with, other regulated cell-death pathways implicated in HCC. Ferroptosis is driven by iron-dependent phospholipid peroxidation and failure of the SLC7A11–glutathione–GPX4 antioxidant system. Its immune consequences are highly dependent on the affected cellular compartment. In HCC models, induction of ferroptosis in tumor-associated macrophages through suppression of SLC7A11 and GPX4 has been reported to inhibit M2-like polarization and promote an antitumor macrophage phenotype [31]. Necroptosis, by contrast, is principally executed through the RIPK1–RIPK3–MLKL pathway and can exert context-dependent immune effects. Although complete necroptotic execution may promote DAMP-mediated immune activation, sublethal necroptotic signaling in hepatocytes can induce the release of CCL20 and MCP-1, activate procarcinogenic monocyte-derived macrophages, and facilitate hepatocarcinogenesis [32]. Necroptosis-related proteins have also been associated with the abundance of intratumoral T cells and clinical outcomes in HCC. These observations illustrate that lytic cell death does not invariably generate an antitumor immune response. Compared with ferroptosis and necroptosis, mitoxyperilysis is initiated by the convergence of innate immune activation and metabolic deprivation and involves prolonged mitochondria–plasma membrane contact, mTORC2-regulated cytoskeletal positioning, and spatially restricted oxidative membrane damage [24]. In the present study, mitoxyperilysis-inducing conditions were associated with increased intracellular and extracellular ANXA1 and an enhanced M2-like macrophage phenotype. These findings suggest a potentially distinct route through which mitochondrial stress in HCC cells may contribute to macrophage reprogramming. Nevertheless, because pathway-specific loss-of-function experiments were not performed, ANXA1–FPR signaling should currently be regarded as a candidate mechanism rather than a definitively established mediator.

Several limitations of the present study should be acknowledged. First, the Mitoxyperilysis-Related Transcriptional Score was derived from bulk transcriptomic data and should be regarded as a mechanism-informed proxy rather than a direct measurement of mitoxyperilysis execution. Its strong association with immune infiltration also indicates that immune and stromal cell composition may partly contribute to the observed prognostic signal. Second, CellChat analysis predicts potential ligand–receptor communication based on gene-expression patterns and does not directly confirm functional ANXA1–FPR signaling. Third, although extracellular ANXA1 was increased under the mitoxyperilysis-inducing condition and ANXA1 overexpression promoted an M2-like macrophage phenotype, ANXA1 knockdown and FPR-blockade experiments were not performed; therefore, the requirement of this pathway remains to be established. In addition, the proteins assessed by Western blotting represent supportive mitochondrial and oxidative-stress-related measurements rather than specific markers of mitoxyperilysis, and concurrent activation of other cell-death or stress pathways cannot be completely excluded. Finally, the experimental findings were obtained using Huh-7 cells and THP-1-derived macrophages in vitro, and further validation in additional cell models, in vivo systems, and prospective clinical cohorts is warranted.

## 5. Conclusions

This study identifies a close association between mitoxyperilysis-related transcriptional features and an immunosuppressive tumor microenvironment in hepatocellular carcinoma. Tumors with relatively higher Mitoxyperilysis-Related Transcriptional Scores exhibited increased immune and stromal infiltration, enhanced immune-checkpoint expression, enrichment of M2-like macrophages, and poorer overall survival in unadjusted analysis. Single-cell communication analysis and experimental validation further supported ANXA1 as an important mediator of macrophage reprogramming. Mitoxyperilysis-inducing conditions increased intracellular and extracellular ANXA1, ANXA1 overexpression promoted M2-like macrophage polarization, and ANXA1 knockdown attenuated this response and reduced tumor burden in vivo. Collectively, these findings suggest that mitoxyperilysis-related tumor-cell stress may facilitate hepatocellular carcinoma progression through ANXA1-associated macrophage polarization. However, the transcriptional score should be interpreted as a mechanism-informed proxy, and direct involvement of formyl peptide receptor signaling requires further validation. The mitoxyperilysis–ANXA1–macrophage axis may provide a potential framework for future biomarker development and therapeutic investigation.

## Data Availability

The publicly available datasets analyzed in this study can be accessed through TCGA-LIHC, GEO accession numbers GSE14520 and GSE149614, and the ICGC Data Portal. The processed single-cell RNA-sequencing data used for the CellChat analysis are available from GEO under accession number GSE149614.

## Supplementary Materials

The following supporting information can be downloaded at https://www.mdpi.com/article/10.3390/cancers18172786/s1: Figure S1: The original Western blot images of Figure 6E,G; Table S1: Antibodies used in the present study.
