# Supplementary material for: Mitoxyperilysis Drives Immunosuppressive Tumor Microenvironment Remodeling and Hepatocellular Carcinoma Progression via ANXA1-Mediated Macrophage M2 Polarization
Source: Cancers (Basel). 2026 Aug 27;18(17):2786. doi: 10.3390/cancers18172786 (PMC13565249; doi:10.3390/cancers18172786)
Supplement: Supplementary file 1 [file cancers-18-02786-s001.zip › cancers-4432757-supplementary.pdf]

Figure 6 E

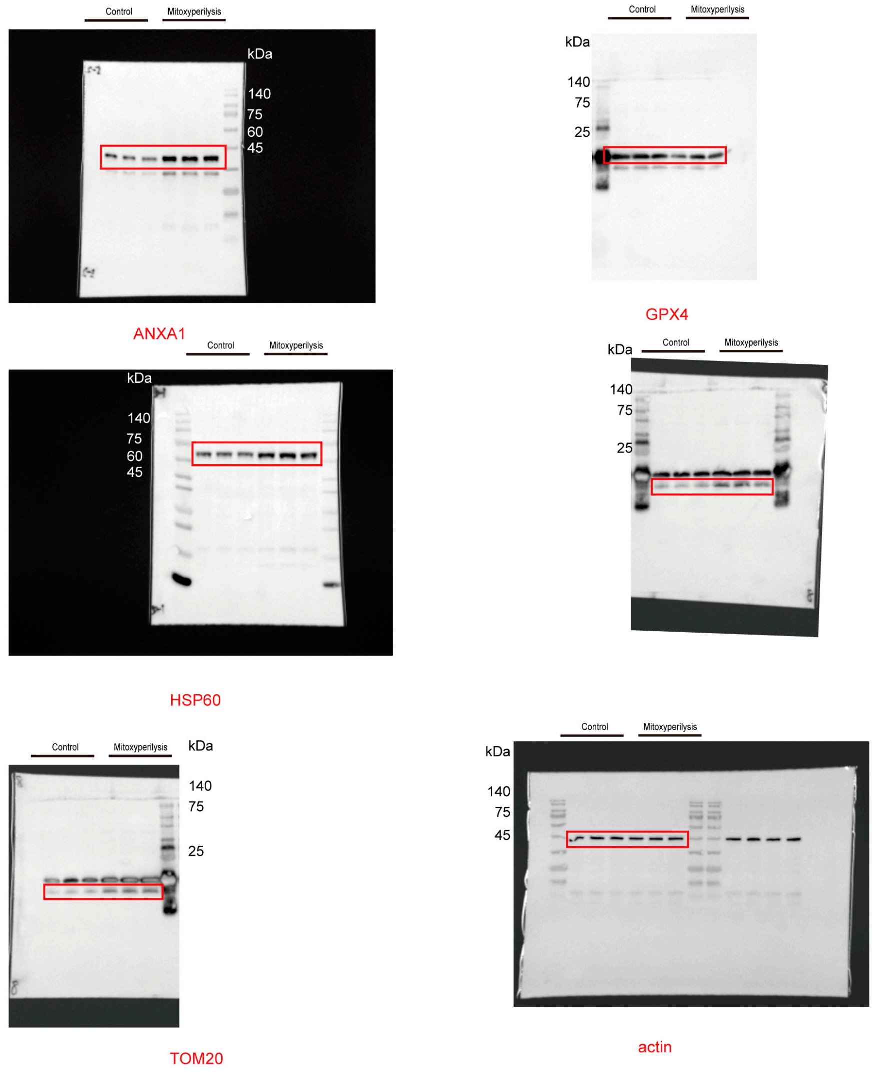

Figure 6 G

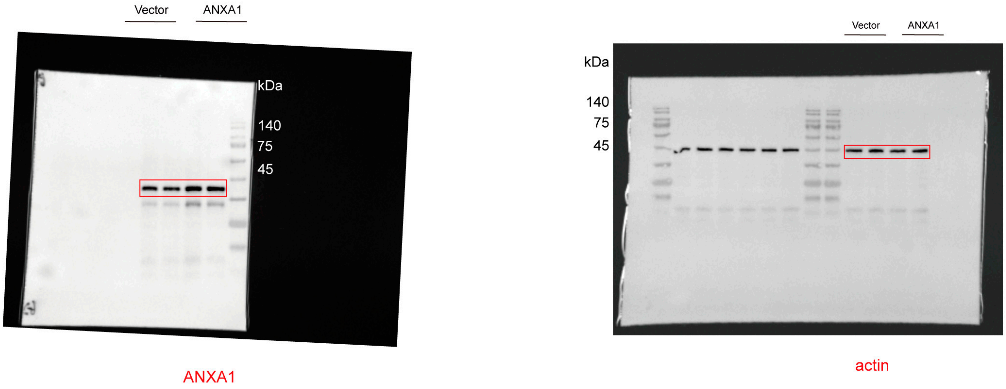

Figure S1. The original Western blot images of Figure 6E,G.

Table S1. Antibodies used in the present study.

Target	Catalog no.	Clone	Host/isotype	Conjugate	Application	Working dilution or amount	Official recommended range	Manufacturer
ANXA 1	21990-1-AP	N/A (polyclonal)	Rabbit IgG	Unconjugated	WB	1:25,000	1:5,000-1:50,000	Proteintech, Wuhan Sanying Biotechnology Co., Ltd., Wuhan, China
GPX4	67763-1-Ig	3F5G5	Mouse IgG	Unconjugated	WB	1:2,000	1:1,000-1:4,000	Proteintech, Wuhan Sanying Biotechnology Co., Ltd., Wuhan, China
HSP60	66041-1-Ig	2F10E7	Mouse IgG1	Unconjugated	WB	1:20,000	1:5,000-1:50,000	Proteintech, Wuhan Sanying Biotechnology Co., Ltd., Wuhan, China
COX IV	66110-1-Ig	2A7B2	Mouse IgG1	Unconjugated	WB	1:20,000	1:5,000-1:50,000	Proteintech, Wuhan Sanying Biotechnology Co., Ltd., Wuhan, China
TOM20	66777-1-Ig	1D6F5	Mouse IgG2b	Unconjugated	WB	1:2,000	1:1,000-1:5,000	Proteintech, Wuhan Sanying Biotechnology Co., Ltd., Wuhan, China
β-actin	66009-1-Ig	2D4H5	Mouse IgG2b	Unconjugated	WB	1:50,000	1:20,000-1:100,000	Proteintech, Wuhan Sanying Biotechnology Co., Ltd., Wuhan, China
Goat anti-mouse IgG (H+L)	SA00001-1	N/A (polyclonal)	Goat IgG	HRP	WB secondary	1:5,000	1:2,000-1:10,000 with conventional ECL	Proteintech, Wuhan Sanying Biotechnology Co., Ltd., Wuhan, China
Goat anti-rabbit IgG (H+L)	SA00001-2	N/A (polyclonal)	Goat IgG	HRP	WB secondary	1:5,000	1:2,000-1:10,000 with conventional ECL	Proteintech, Wuhan Sanying Biotechnology Co., Ltd., Wuhan, China

Target	Catalog no.	Clone	Host/isotype	Conjugate	Application	Working dilution or amount	Official recommended range	Manufacturer
CD206	81525-1-RR	1G15	Rabbit IgG, recombinant monoclonal	Unconjugated	IF/ICC	1:200	1:50-1:500	Proteintech, Wuhan Sanying Biotechnology Co., Ltd., Wuhan, China
Goat anti-rabbit IgG (H+L)	SA00013-2	N/A (polyclonal)	Goat IgG	CoraLite®488	IF secondary	1:500	1:100-1:1,000	Proteintech, Wuhan Sanying Biotechnology Co., Ltd., Wuhan, China
CD163	PE-65169	GHI/61	Mouse IgG1, κ	PE	Flow cytometry	5 μL/10^6 cells in 100 μL	5 μL/10^6 cells in 100 μL	Proteintech, Wuhan Sanying Biotechnology Co., Ltd., Wuhan, China
CD206	APC-65155	15-2	Mouse IgG1, κ	APC	Flow cytometry	5 μL/10^6 cells in 100 μL	5 μL/10^6 cells in 100 μL	Proteintech, Wuhan Sanying Biotechnology Co., Ltd., Wuhan, China

Abbreviations: WB, Western blotting; IF/ICC, immunofluorescence/immunocytochemistry; HRP, horseradish peroxidase; PE, phycoerythrin; APC, allophycocyanin; ECL, enhanced chemiluminescence; N/A, not applicable.
